# *In Vitro* Metabolic Stability of Exendin-4: Pharmacokinetics and Identification of Cleavage Products

**DOI:** 10.1371/journal.pone.0116805

**Published:** 2015-02-27

**Authors:** Sha Liao, Yuanjun Liang, Zhiwei Zhang, Jinglai Li, Juan Wang, Xiaoying Wang, Guifang Dou, Zhenqing Zhang, Keliang Liu

**Affiliations:** 1 Beijing Institute of Pharmacology and Toxicology, 27 Taiping Road, Haidian District, Beijing, 100850, PR China; 2 Beijing Institute of Transfusion Medicine, 27 Taiping Road, Haidian District, Beijing, 100850, PR China; Chang-Gung University, TAIWAN

## Abstract

The aim of this study was to investigate the metabolic stability and cleavage sites of exendin-4 in rat tissue homogenates, as well as to identify the types of proteases involved in exendin-4 degradation. The stability of exendin-4 in kidney and liver homogenates from rats was evaluated using liquid chromatography-electrospray ionization mass spectrometry (LC-ESI-MS) with gradient elution. Furthermore, we used a combination of matrix-assisted laser desorption/ionization time-of-flight mass spectrometry (MALDI-TOF MS) and LC-ESI-MS/MS to identify the structures of the major degradation products of exendin-4, and peptidase inhibitors were used to characterize exendin-4 degradation in rat liver and kidney homogenates and to identify the proteases involved in exendin-4 metabolism. Exendin-4 had a half-life of 7.8 and 100.9 min in the kidney and liver homogenate, respectively. The enzymes most likely to be involved in the degradation of exendin-4 were aminopeptidases, serineproteases, and metalloproteases. Exendin-4(15-39) and exendin-4(16-39) were the predominant direct exendin-4 metabolites in the kidney, and the main product of exendin-4 metabolism in the liver was exendin-4(12-39). Our results indicated that the metabolism of exendin-4 involved an initial endoproteolytic cleavage and subsequent exoproteolytic digestion. The degradation of exendin-4 in the kidney and liver homogenates followed distinct patterns, and the primary cleavage sites of exendin-4 degradation in rat kidney homogenates were located after AA-14, and -15, whereas those in rat liver homogenates were located after AA-11.

## Introduction

In recent years, the significance of the pharmacokinetic profile of a drug as a determinant of its *in vivo* action has attracted increased attention, and its potential as a tool to expedite drug research and development has been exploited [1]. Although pharmacokinetic principles have been widely used in the development of conventional low-molecular-weight drugs, a large number of studies have investigated the metabolic stability and biological processing of therapeutic polypeptides [2]. During systemic circulation of peptides, fast renal clearance and degradation by enzymes in the blood, liver, and kidney [3] often result in short plasma half-lives, which limit their therapeutic activity. To improve the efficacy of therapeutic polypeptides, research efforts are aimed at thorough characterization of the metabolic processing of peptides and establishment of quantitative structure-stability relationships (QSSRs) that can be used to design peptide-based therapeutics with prolonged metabolic stability and improved pharmacokinetic profiles [4].

In 2003, the International Diabetes Federation (IDF) estimated that almost 200 million people around the world had diabetes mellitus (DM). Approximately 90–95% of people with DM have type 2 diabetes mellitus (T2DM), which is characterized by hyperglycemia, insulin resistance, absolute or relative insulin deficiency, hyperglucagonemia, and increased hepatic glucose production [5,6]. An optimal therapy for controlling the circulating glucose levels has not been established, and many currently available therapies are associated with unfavorable side effects, which limit their use [7]. Thus, novel therapeutic approaches for glycemic control that complement existing therapies and preserve the normal physiological response to caloric intake are urgently required. One such approach to glycemic control is based on the action of glucagon-like peptide 1 (GLP-1). GLP-1 is an incretin hormone that is released from the L cells of the distal intestine in response to nutrients. GLP-1 possesses a number of beneficial anti-diabetic properties and may promote β-cell preservation and improve neogenesis [8,9]. However, the usefulness of native GLP-1 is limited by its metabolic instability. Circulating GLP-1 undergoes rapid proteolytic cleavage by rat and human plasma dipeptidyl peptidase-IV (DPP-IV)[10], and biologically active GLP-1 has an apparent serum half-life of only 1–2 min in humans [11]. Therefore, long-acting agonists of the GLP-1 receptor have been developed.

**Fig 1:**
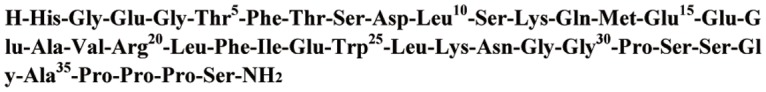
The amino acid sequence of exendin-4.

Exendin-4, a 39-amino acid peptide that shares 53% amino acid sequence homology with GLP-1, is resistant to cleavage by plasma DPP-IV, and thus has a longer half-life and duration of action, as well as greater potency *in vivo* [11]. On the basis of the results of preclinical and clinical trials, Byetta, a synthetic version of exendin-4 (Fig. 1), was approved by the U.S. Food and Drug Administration in April 2005 for adjunctive glycemic control in patients with T2DM who are taking metformin, a sulfonylurea, or a combination of metformin and a sulfonylurea [12,13]. Although naturally resistant to plasma DPP-IV, Edwards et al. [14] reported that the serum half-life of exendin-4 was only 26 min in humans after intravenous (i.v.) infusion. Moreover, additional pharmacokinetic (PK) studies after i.v. administration of exendin-4 using animal models (rat, pig, and monkey) [15–17] show rapid elimination of exendin-4 *in vivo* (biological half-life [t_1/2b_], 18–41 min). In contrast, when a mechanism-based pharmacokinetics (PK) and pharmacodynamics (PD) model was used to assess the role of exendin-4 in the regulation of the glucose and insulin system, it was found that both the plasma drug concentration (C) and the drug-receptor complex (RC) acted as driving forces for its insulinotropic effect, which persisted for more than 300 min [18]. The short plasma half-life, high potency, and long *in vivo* duration of effect of exendin-4 indicated that evaluating its peripheral metabolism would provide information that might illuminate its role in glycemic control. Detailed distribution studies indicated that the short elimination half-life of exendin-4 is primarily due to the degradation of the peptide in the liver and kidney, which are the major target organs for pharmacological activity and metabolism/elimination of exendin-4 in humans, rats, and pigs *in vivo* [19–21]. Further studies showed rapid and extensive metabolism of exendin-4; exendin-4 was mainly eliminated via the kidneys by peritubular uptake and subsequent re-absorption and intracellular catabolism [16,22], indicating that it remained prone to renal elimination, similar to other low-molecular-weight peptides (molecular weight, approximately 3.4 kDa) [23].

In addition, Goke et al. [24,25] observed that GLP receptor-mediated endocytosis appears to be a general mechanism by which exendin-4 is internalized from the cell surface into the intracellular matrix after initial cell surface binding. After it is taken up into the cell, exendin-4 is subjected to hydrolysis by soluble and membrane-bound enzymes, which results in its inactivation. These findings indicate that further detailed characterization of the proteolytic processes acting on exendin-4 at an intracellular level will provide information relevant to its peripheral metabolism and illuminate the influence of exendin-4 metabolism on its regulation of the glucose and insulin system. Furthermore, in depth characterization of tissue-specific peptide degradation will support efforts aimed at increasing peptide stability against the action of peptidases, as well as facilitate a higher degree of targeting to specific tissues, and thus support the development of effective pharmacological preparations on the basis of these peptides [26,27]. Limited information is available concerning the metabolism of exendin-4 by peptidases from rat tissues.

Therefore, in this study, we characterized the *in vitro* metabolic stability of exendin-4 in rat liver and kidney homogenates. Furthermore, we used a combination of liquid chromatography-electrospray ionization mass spectrometry (LC-ESI-MS/MS) and matrix-assisted laser desorption/ionization time-of-flight mass spectrometry (MALDI-TOF MS) to identify the major degradation products of exendin-4. In addition, we characterized the effects of different types of standard protease inhibitors on the stability of exendin-4 to identify enzymes involved in its metabolism.

## Materials and Methods

### Animal homogenates and materials

All experimental protocols were approved by the Animal Ethics Committee of the Beijing Institute of Pharmacology and Toxicology (No. 11400700031602). Six male Sprague-Dawley rats weighing 190–210 g were purchased from Beijing Vital River Laboratories (Beijing, China). All animal experiments were conducted in the Beijing Center for Drug Safety Evaluation according to protocols that were in agreement with the guidelines of the Institutional Animal Care and Use Committee of the Center, which complied with the guidelines of the Association for Assessment and Accreditation of Laboratory Animal Care International (AAALAC). Before initiation of the study, the animals were starved overnight with free access to water, and on the next morning they were anesthetized with 10% chloral hydrate (3 mL/kg intraperitoneally), decapitated, and bled. The liver and kidney were isolated from each rat, washed in ice-cold saline solution (0.9% sodium chloride), and trimmed of adhering tissue. The excised tissue was weighed, cut into small pieces, and 4 mL of ice-cold incubation buffer (50 mM Tris-HCl, pH 7.4) was added per gram of tissue. For each tissue type, samples from the 6 rats were pooled and homogenized 3 times by using a Teflon digital homogenizer (15 s each time). The solution was allowed to cool on ice between each homogenization step. The homogenized solution was centrifuged (2000 *g*) for 10 min at 4°C, and the supernatants were stored at -80°C until further analysis. *In vitro* assays were performed within 3 days of tissue collection. The total protein concentration in the tissue homogenates was determined using the bicinchoninic acid (BCA) colorimetric assay (Pierce Biotechnology, Rockford, IL, USA) using bovine serum albumin as a reference standard. Homogenates were diluted to 2.5 mg protein/mL as required.

### Materials

Exendin-4 peptide (4186.6 Da) was synthesized by the Beijing Institute of Pharmacology and Toxicology, purified using high-performance liquid chromatography (HPLC) (>99% purity), and stored at -80°C. Acetonitrile, methanol, formic acid (99%, HPLC grade), and analytical-grade trifluoroacetic acid (TFA) were obtained from Sigma-Aldrich (St Louis, MO, USA). HPLC-grade water was doubly purified with a Milli-Q system (Millipore, Molsheim, France). All peptidase inhibitors used in this study were obtained from Merck KGaA (Darmstadt, Germany). All other reagents were of the highest grade that was commercially available at the time of the study.

### Degradation of exendin-4 in tissue homogenates

Degradation of exendin-4 was measured after it was incubated with rat kidney and liver homogenates. Ice-cold incubation buffer (50 mM Tris-HCl, pH 7.4) was added to the diluted tissue homogenate, the resulting mixture was vortexed, and the reaction was initiated by the addition of exendin-4 at a final concentration of 10 μM. Samples (50 μL) were withdrawn at different incubation time points (0, 5, 10, 15, 30, 45, 60, 90, 120, 240, and 360 min) from a water bath at 37°C, mixed with 3 volumes of ice-cold acetonitrile to terminate the enzymatic activity, and vortexed [28]. The mixtures were centrifuged at 10,000 *g* for 10 min at 4°C, and the resulting supernatants were evaporated using Speed-Vac (Eppendorf, Hamburg, Germany) and stored at -40°C until further analysis. The dried residue was re-dissolved in 50 μL of the HPLC mobile phase and analyzed using LC-ESI-MS and LC-ESI-MS/MS to characterize stability and identify metabolites, respectively. Half-life decay rates were derived by fitting a non-linear Michaelis-Menten model to the measured metabolic stability as a function of time.

For MALDI-TOF MS analysis, a 1-μL aliquot of the sample solution was applied on the target, and 1 μL of a freshly prepared saturated solution (a-cyano-4-hydroxy-cinnamic acid in acetonitrile/0.1% trifluoroacetic acid in water) of matrix was added and subjected to the MALDI-MS target for mass spectral analysis. Appropriate blanks, controls (i.e. no protein), and reference solutions were prepared.

### Inhibition of exendin-4 metabolism

To identify the types of proteolytic enzymes involved in the metabolism of exendin-4, the peptide was incubated in rat liver and kidney homogenates in the presence of various general and specific enzyme inhibitors. We examined the abilities of different concentrations of the following protease inhibitors to increase exendin-4 metabolic stability: *N*-ethylmaleimide (NEM) (cysteine protease inhibitor, 10 mM), phenanthroline (metalloprotease inhibitor, 2 mM), PPACK (DPP-IV/thrombin inhibitor, 210 μM), soybean (trypsin inhibitor, 1 mg/mL), DL-thiorphan (endopeptidase 24.11 inhibitor, 2 mM), captopril (angiotensin-converting enzyme inhibitor, 30 μM), diisopropylfluorophosphate (DFP/serine protease inhibitor, 10 mM), phenylmethylsulfonyl fluoride (PMSF/serine protease inhibitor, 1 mM), amastatin (aminopeptidase inhibitor, 100 μM), and bestatin (aminopeptidase inhibitor, 2 mM). Protease inhibitors were individually added to diluted homogenates, vortexed, and pre-incubated for 10 min at 37°C, after which exendin-4 was added to achieve the concentration listed above. Each pre-treated biological matrix sample was incubated with exendin-4 (final concentration, 10 μM) for an additional 30 min (kidney) or 60 min (liver) at 37°C and compared to similar preparations containing no enzyme inhibitor, as well as to synthetic peptide incubated for 0 min. Samples were treated and analyzed as described above. Values of percentage inhibition of peptide degradation were calculated using the following formula:

[k (without inhibitor) - k (with inhibitor)]/k (without inhibitor) ×100

### Mass spectrometry

HPLC experiments were performed using a Finnigan SurveyorHPLC system (Thermo Electron, San Jose, CA, USA). A triple-stage quadrupole mass spectrometer (TSQ Quantum Discovery; Thermo Electron) equipped with an ESI source was used to detect intact exendin-4. LC-MS was performed in the positive mode and selected ion monitoring (SIM) mode. The Finnigan SurveyorHPLC system and Finnigan TSQ Quantum Discovery were controlled using Xcalibur version 1.4 software. Data acquisition was performed with Xcalibur 1.4 software (ThermoFinnigan, San Jose, CA, USA), and peak integration and calibration were performed using LCQuan software (ThermoFinnigan). The other MS parameters were as follows: ESI needle voltage, 4300 V; nitrogen sheath gas pressure, 45 arb units; auxiliary gas pressure, 10 arb units; ion transfer capillary temperature, 250°C; tube lens offset, -35 V (at *m/z* 838); and source collision-induced dissociation (CID) offset, 20 V. Separation of exendin-4 was achieved using an SB300C18 column with an internal diameter (i.d.) of 100 mm × 2.1 mm (particle size, 3.5 μm, Agilent, Santa Clara, CA, USA) packed with on-line filters. The mobile phases were (A) 0.1% (v/v) formic acid (FA) in water and (B) acetonitrile. Samples were eluted using a timed, linear gradient from 15% B at t = 0.0 min to 70% B at t = 10.0 min. After a hold of 1 min at 70% B, the column was recycled to 15% B and re-equilibrated for 10 min. The run-time was 30 min per injection. The flow rate was 0.2 mL/min. The injection volume for all LC experiments was 15 μL. A ThermoFinnigan LCQ mass spectrometer was used to analyze the degradation products of exendin-4 by evaluation of product ion spectra derived from the degradation fragments. The residue was re-dissolved in a small volume of the HPLC mobile phase and further analyzed by LC-ESI-MS/MS using the data-dependent scan mode to detect and identify the metabolites (scanning range, *m/z* = 300–1700). The LC-MS operating conditions described above were used with the following modification of the elution method: each sample was eluted with a linear gradient of mobile phase B (formic acid/acetonitrile, 0.1% v/v) from 0 to 70% over a 45-min period. The XCalibur software component Bioworks (3.3.1, ThermoFinnigan) was used to search for the peptide fragments in the ions of each peak.

In addition, metabolites obtained from the degradation of exendin-4 were analyzed using the mass spectrum of MALDI performed in the linear positive ion mode on a Bruker REFLEX III MALDI-TOF mass spectrometer (Bruker-Franzen, Bremen, Germany). The sample (1–2 μL) was mixed with an equal volume of matrix aliquot solution (a-cyano-4-hydroxycinnamic acid) using the dried-droplet technique. Bradykinin, human secretin, and ubiquitin peptide standards (Sigma-Aldrich, St. Louis, MO, USA) were used as standard for external mass calibration of the mass spectrometer.

### Statistical analysis

Results from individual experiments are presented as the mean ± standard error of the mean. Results from peptide degradation experiments are plotted as graphs of the amount of the remaining peptide (area %) versus time. All results are the average of at least 3 experiments performed in triplicate. The statistical significance was determined using analysis of variance (ANOVA) followed by Student-Newman-Keuls test. Differences were considered to be significant at P < 0.05.

## Results

### Stability of exendin-4 in different tissue homogenates of rats

**Fig 2:**
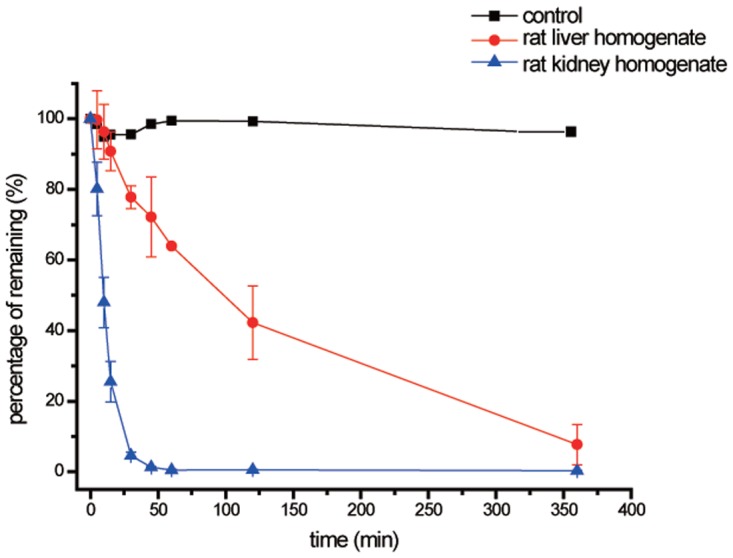
Stability of exendin-4 in rat liver and kidney homogenates. Residual exenatide content at 0 min was considered 100%, and homogenates without exenatide solutions were used as control samples. n = 3.

The metabolic stability of exendin-4 in homogenates of rat liver and kidney tissues is shown in Fig. 2. Exendin-4 was monitored over a period of 360 min in each matrix. After 30 min, approximately 75% of the exendin-4 peptide at the starting point remained in the liver homogenates, whereas exendin-4 was almost completely degraded within 30 min (approximately 5.7% remaining) in the kidneys; no degradation was observed in control experiments over the 360-min incubation period. The half-life of exendin-4 in each matrix was calculated from the non-linear Michaelis-Menten model to determine the stability profile (Table 1). In the presence of rat liver homogenate, exendin-4 was degraded with a decay rate corresponding to a half-life of 100.9 min, while the degradation of exendin-4 was more than 10-fold faster in the kidney, where its half-life was 7.8 min.

**Table 1:** Degradation of exendin-4 in rat tissue homogenates at pH 7.4 and 37°C.

Tissue homogenate	Half-lives (min)
kidney	7.8 ±0.58
liver	100.9 ± 10.2

The rate constants of degradation were estimated by the non-linear Michaelis-Menten model. The data collected at 0 min after starting the incubation are presented as the initial rate of metabolism.

### Use of protease inhibitors to stabilize exendin-4

**Fig 3:**
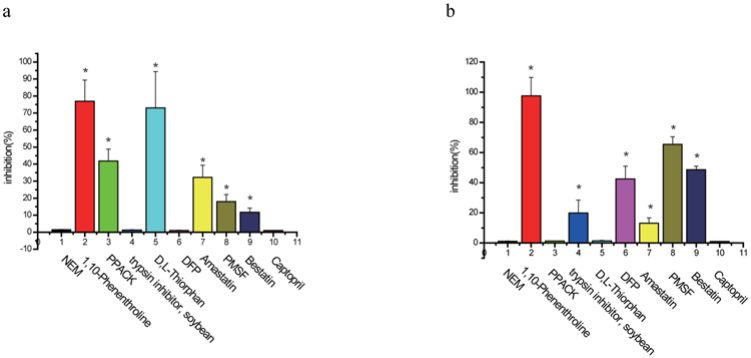
Effect of protease inhibitors on exendin-4 degradation. Inhibition of exendin-4 degradation was measured in rat (a) kidney and (b) liver pre-treated with the indicated protease inhibitor. The amount of peptide remaining was assayed after 30 min (kidney) or 60 min (liver). The results are expressed as the percentage inhibition of exendin-4 degradation. n = 3; *, P < 0.05 compared to the control group.

To identify the types of proteolytic enzymes involved in exendin-4 metabolism, we screened various protease inhibitors (Fig. 3) to determine their ability to inhibit exendin-4 degradation in rat tissue homogenates over a 30-min (kidney, Fig. 3a) or 60-min (liver, Fig. 3b) period. Tissue homogenates treated with 1,10-phenanthroline showed the greatest extent of exendin-4 degradation (>77%), and the degradation level was similar in kidney in liver homogenates. In addition, amastatin, PMSF, and bestatin inhibited exendin-4 degradation in liver and kidney homogenates, but in a tissue-specific manner; PMSF and bestatin were more effective in the liver homogenates (65% and 48%, respectively) than in the kidney homogenates (18% and 11%, respectively), but amastatin showed more effectively inhibited exendin-4 metabolism in the kidney (32%) than in the liver (13.2%). PPACK and DL-thiorphan had a significant inhibitory effect on exendin-4 degradation in the kidney (41% and 73%, respectively), but these inhibitors had no effect on exendin-4 degradation in the liver. In contrast, the trypsin inhibitor (soybean) and DFP inhibited the metabolism of exendin-4 by 20% and 42%, respectively, in the liver, but these compounds had no effect in the kidney. Exendin-4 degradation was not inhibited by NEM or captopril. The protease inhibitor results are summarized in Fig. 3.

### Identification of exendin-4 degradation products in rat tissue homogenates

**Fig 4:**
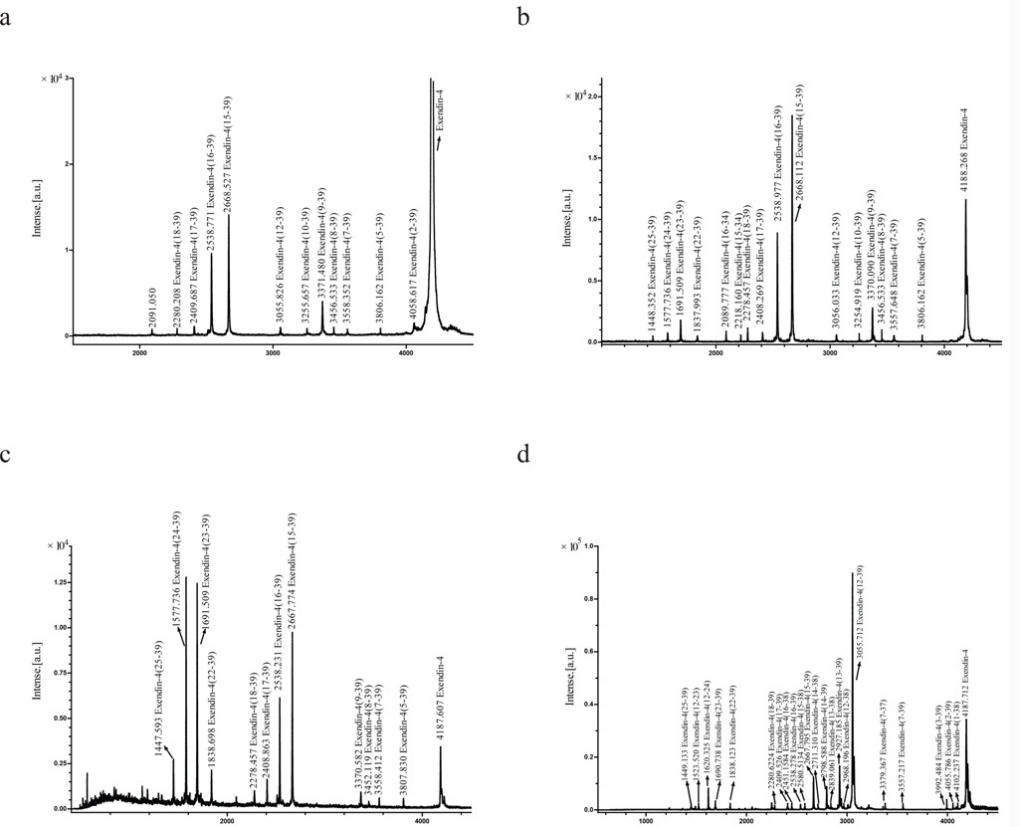
Matrix-assisted laser desorption/ionization time-of-flight mass spectrometry (MALDI-TOFMS) spectra of degradation products from exendin-4 incubated in rat kidney homogenate for (a) 5 min, (b) 15 min, and (c) 30 min, and in rat liver homogenate for (d) 6 h.

**Fig 5:**
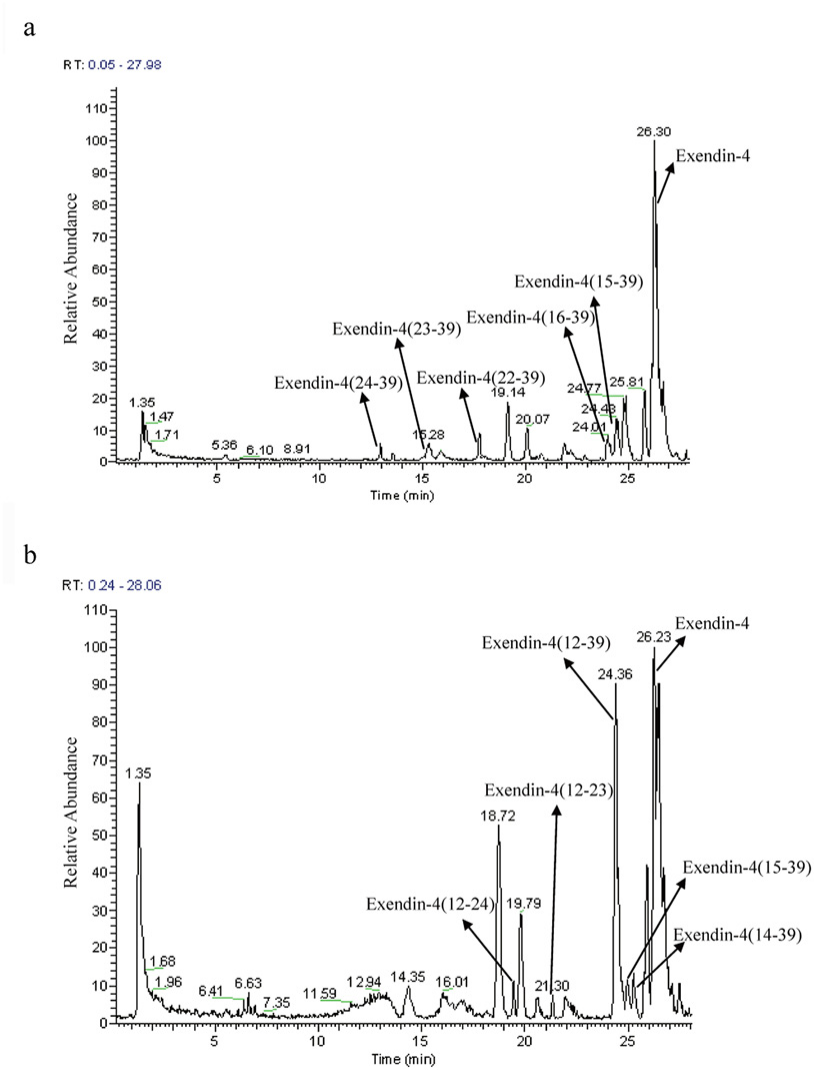
Liquid chromatography-electrospray ionization mass spectrometry (LC-ESI-MS) chromatographic profile of exendin-4 metabolites after incubation in (a) rat kidney homogenate for 15 min and (b) in liver homogenate for 6 h.

MALDI-TOF MS analysis was performed by direct analysis of samples containing metabolites emerging from the degradation of exendin-4 in contact with the rat kidney homogenate. From the results obtained after 5, 10, 15, 30, 45, 60, 90, 120, 240, and 360 min, 3 typical MALDI-TOF MS spectra are shown in Fig. 4a, b, and c. Similar to the findings of the stability studies, the degradation fragments of exendin-4 were observed mainly within 60 min from the initiation of the incubation of the rat kidney homogenate *in vitro*. After 5 min of incubation, the C-terminal metabolites exendin-4(15–39), exendin-4(16–39), exendin-4(17–39), and exendin-4(18–39) were detected. Moreover, the fragments exendin-4(2–39), exendin-4(5–39), exendin-4(7–39), exendin-4(8–39), exendin-4(9–39), exendin-4(10–39), and exendin-4(12–39) were monitored without their N-terminal counterpart (Fig. 4a). After 15 to 30 min of incubation, additional fragments such as exendin-4(22–39), exendin-4(23–39), exendin-4(24–39), and exendin-4(25–39) were detected, whereas their respective N-terminal counterparts remained undetectable (Fig. 4b and 4c). Although initially present from 5 to 30 min after incubation, the 2 early and major degradation products exendin-4(15–39) and exendin-4(16–39) were not detected after 60 min (S1 Fig.), which suggested that they were further degraded. In addition, the presence of these two major fragments was confirmed by LC-ESI-MS/MS (Fig. 5a). In addition, exendin-4(15–34) and exendin-4(16–34), which can be detected by MALDI-TOF MS after 15 min of incubation with the rat kidney homogenate as determined by MALDI-TOF MS (Fig. 4b), may have originated from the degradation of the early major degradation products exendin-4(15–39) and exendin-4(16–39) by carboxypeptidases or endopeptidases.

**Fig 6:**
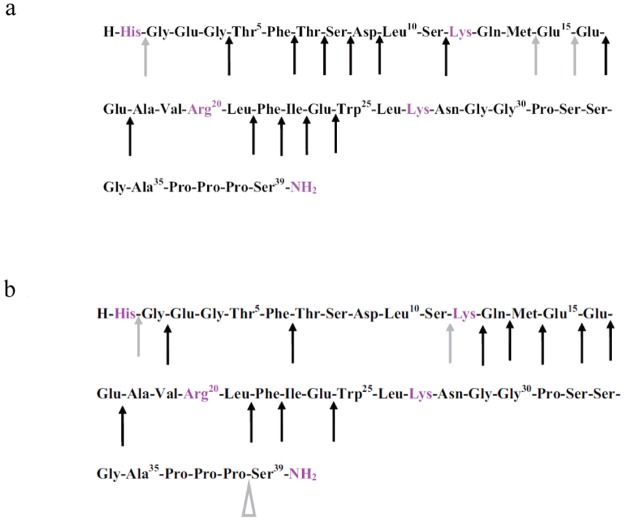
Proposed schemes for the metabolic cleavage of exendin-4 in rat (a) kidney and (b) liver homogenates. Arrowheads indicate cleavage sites in exendin-4: solid arrowheads, sites of concomitantly detected N- and C-terminal counterparts; open arrowheads, only N-terminal fragment detected; up arrowheads, only C-terminal fragment detected. Grey symbols indicate cleavage sites that are targeted at the onset of incubation. Black symbols indicate cleavage sites that are targeted in major metabolites or intact exendin-4 by sequential cleavage via aminopeptidase or carboxypeptidase activity.

A summary of the suggested cleavage sites of exendin-4 by proteases present in rat kidney homogenate are shown in Fig. 6a. An initial cleavage seems to occur at 3 different sites, namely between the residues His^1^-Gly^2^, Met^14^-Glu^15^, and Glu^15^-Glu^16^, as observed in the C-terminal fragment. Subsequently, further truncations of initial metabolites or intact exendin-4 occurred by sequential cleavage via aminopeptidase or carboxypeptidase activity, in which exendin-4(2–39), exendin-4(5–39), exendin-4(7–39), exendin-4(8–39), exendin-4(9–39), exendin-4(1–23), and exendin-4(12–39) were cleaved from intact exendin-4 and exendin-4(17–39), and exendin-4(18–39), exendin-4(22–39), exendin-4(23–39), exendin-4(24–39), and exendin-4(25–39) were cleaved from exendin-4 (15–39) and exendin-4(16–39). The resulting cleavage pattern is shown in Fig. 6a. A summary of the measured values of the molecular masses corresponding to the different metabolites in given in Table 2.

**Table 2:** Matrix-assisted laser desorption/ionization time-of-flight mass spectrometry (MALDI-TOF MS) peak spectrum of kidney (15 min) homogenate metabolites, (M +H)^**+**^, of exendin-4.

Peak no.	MALDI	ESI	Fragments
1	1448.4		25–39
2	1577.7	1578.7	24–39
2	1691.5	1691.8	23–39
3	1838.0	1839.1	22–39
4	2089.8		16–34
5	2218.2		15–34
6	2278.5		18–39
7	2408.3		17–39
8	2511.3		1–22
9	2539.0	2537.2	16–39
10	2668.1	2665.8	15–39
11	3055.0		12–39
12	3254.9		10–39
13	3370.1		9–39
14	3386.2		
15	3452.1		8–39
16	3557.6		7–39
17	3806.1		5–39
18	4188.3	4185.6	1–39

When exendin-4 was incubated with the rat liver homogenate, it was degraded into a total of 23 C-terminal metabolites (Fig. 4d). Samples were directly analyzed using MALDI-TOF MS, and the resulting cleavage pattern is shown in Fig. 6b. After 10 min of incubation, a cleavage site was found between the residues Ser^11^-Lys^12^, as determined by the occurrence of a C-terminal. After periods ranging from 10 to 360 min of incubation, additional fragments such as exendin-4(1–38), exendin-4(2–39), exendin-4(3–39), exendin-4(7–39), exendin-4(12–38), exendin-4(13–39), exendin-4(13–38), exendin-4(14–39), exendin-4(14–38), exendin-4(15–39), exendin-4(15–38), exendin-4(16–39), exendin-4(16–38), exendin-4(17–39), exendin-4(18–39), exendin-4(22–39), exendin-4(23–39), and exendin-4(25–39) were found, but without their respective N- and C-terminal counterparts. A summary of the measured values of the molecular masses corresponding to the different metabolites is given in Table 3. A summary of the suggested cleavage sites of exendin-4 by proteases present in rat liver homogenates are shown in Fig. 6b.

**Table 3:** Matrix-assisted laser desorption/ionization time-of-flight mass spectrometry (MALDI-TOF MS) peak spectrum of liver (6 h) homogenate metabolites, (M +H)^**+**^, of Exendin-4.

Peak no.	MALDI	ESI	Fragments
1	1231.3		20–30
2	1362.9		21–32
3	1449.1		**25–39**
4	1460.2		22–35
5	1491.8	1492.3	12–23
6	1620.3	1621.1	12–24
8	1690.7		**23–39**
9	1806.0		**12–25**
10	1838.1		**22–39**
11	1926.1		1–17
12	2056.4		3–20
13	2091.9		13–29
14	2251.0		1–20
16	2280.6		**18–39**
17	2409.5		**17–39**
18	2451.2		**16–38**
19	2538.3		**16–39**
20	2580.5		**15–38**
21	2667.8	2665.9	**15–39**
22	2711.3		**14–38**
23	2798.6	2796.8	**14–39**
24	2815.0		6–28
25	2839.1		**13–38**
26	2927.2		**13–39**
27	2941.7		1–25
28	2968.2		**12–38**
29	3008.8		8–34
30	3055.7	3053.2	**12–39**
31	3070.8	3069.4	10–37
32	3198.2		6–33
33	3379.4		**7–37**
34	3557.2		**7–39**
35	3992.5		**3–39**
36	4055.8		**2–39**
37	4102.2		**1–38**
38	4187.7	4185.6	1–39

The initial exendin-4 concentration of the respective metabolism experiment was 10 μM. The incubation time was 45 min. For comparison, the data of the corresponding electrospray ionization mass spectrometry (ESI-MS) experiment is also included.

The MALDI-TOF MS analysis showed that the major cleavage site of exendin-4 in the rat liver homogenate was Ser11-Lys12 (Fig. 4d), which was indicative of endopeptidase activity. Other fragments seemed to originate from sequential cleavage of the major metabolites by aminopeptidase or carboxypeptidase activity: Glu^3^-Ser^39^NH2 and Thr^7^-Ser^39^NH2 from Gly^2^-Ser^39^NH_2_, Lys^12^-Pro^38^, Gln^13^-Ser^39^NH_2_, Met^14^-Ser^39^NH_2_, Glu^15^-Ser^39^NH_2_, Glu^16^-Ser^39^NH_2_, Glu^17^-Ser^39^NH_2_, Ala^18^-Ser^39^NH_2_, Phe^22^-Ser^39^NH_2_, Ile^23^-Ser^39^NH_2_, and Trp^25^-Ser^39^NH_2_ from Lys^12^-Ser^39^NH_2_, Gln^13^-Pro^38^ from Gln^13^-Ser^39^NH_2_, Met^14^-Pro^38^ from Met^14^-Ser^39^NH_2_, Glu^15^-Pro^38^ from Glu^15^-Ser^39^NH_2_, and Glu^16^-Pro^38^ from Glu^16^-Ser^39^NH_2_.

**Fig 7:**
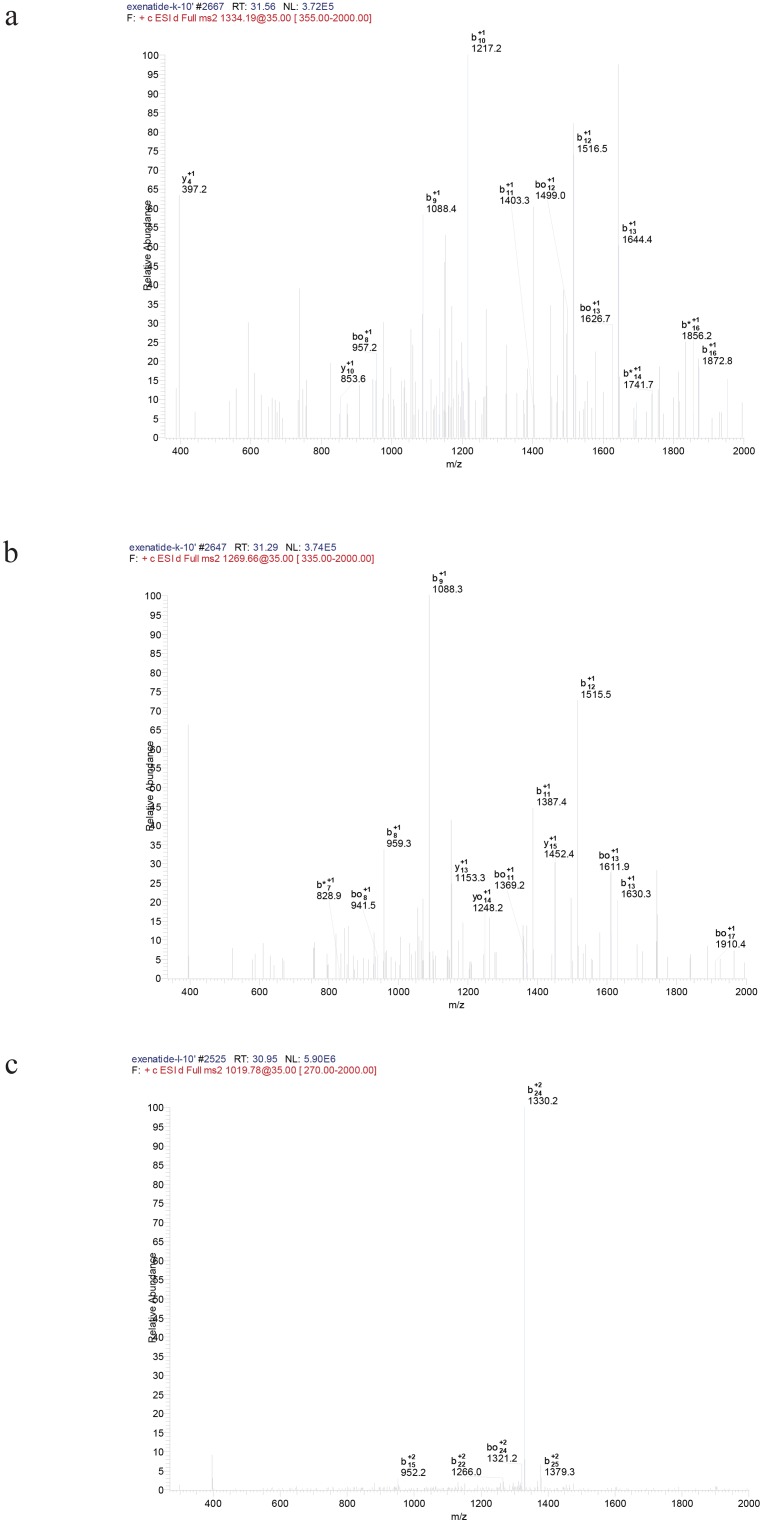
Tandem collision-induced dissociation (CID) mass spectra of doubly protonated peptide (a) exendin-4(15–39), (b) exendin-4(16–39), and (c) exendin-4(12–39). Precursor ions were generated by electrospray ionization (ESI) using the on-line liquid chromatography (LC)-ESI/tandem mass spectrometry (MS-MS) procedure described in the Methods section.

**Fig 8:**
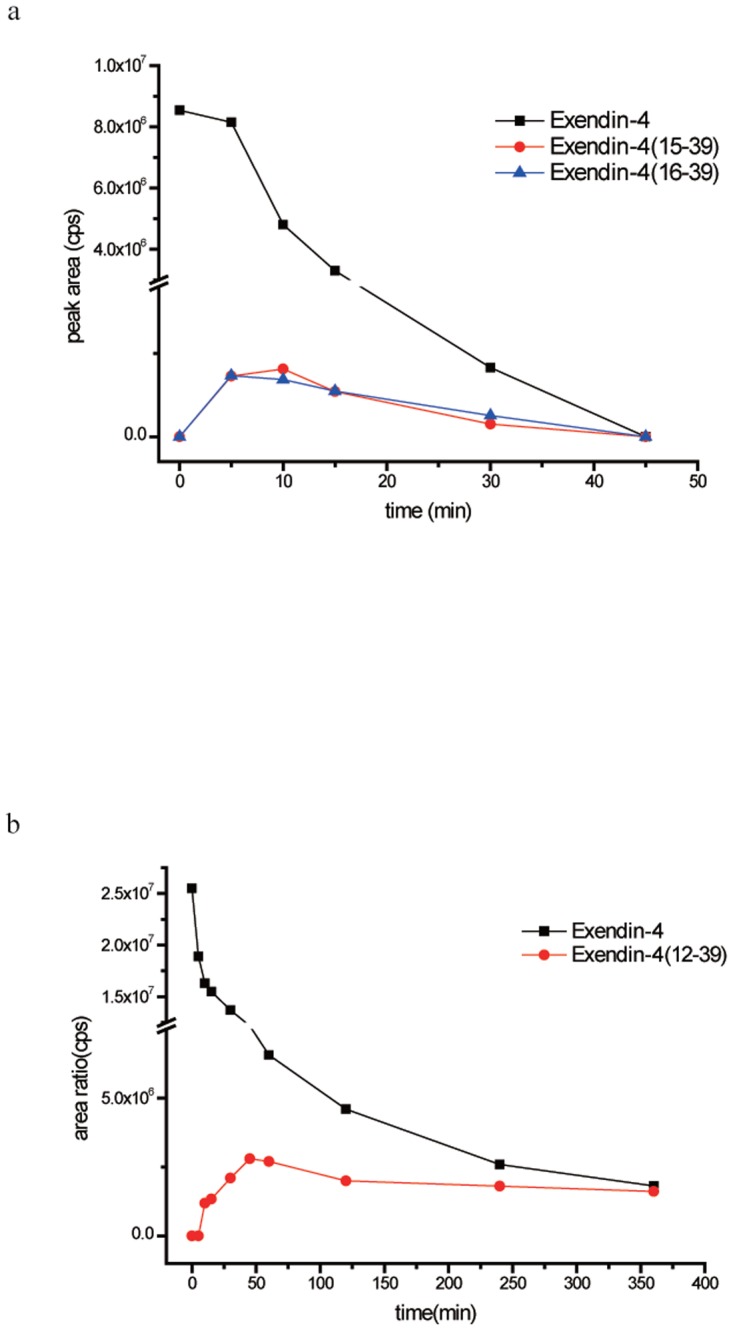
Time course of exendin-4 degradation in (a) rat kidney homogenate and (b) rat liver homogenate in vitro. n = 3.

Representative chromatograms of exendin-4 degradation products in the kidney and liver homogenates after incubation for 15 min and 6 h are shown in Fig. 5a and b, respectively (major products shown), and the CID spectra of the major degradation fragments of exendin-4 in the rat kidney and liver homogenates are shown in Fig. 7a, b, and c. The metabolites showing (M+H)^+^ with *m/z* 2666.1 and 2536.9 were identified as exendin-4(15–39) and exendin-4(16–39) peptide fragments, respectively (Fig. 7a and b); the fragment exendin-4 (12–39) was ruled out from the series b15, b22, b24, and b25 (Fig. 7c). Other metabolites of exendin-4 were confirmed in this manner, and their amino acid sequences were determined using LC-ESI-MS/MS. This procedure was used to assign the degradation fragments (Table 2 and 3). A summary of the metabolic fate of exendin-4 after exposure to the rat kidney and liver homogenates, as determined by LC-ESI-MS/MS, is shown in Fig. 8a and b. Exendin-4 was degraded over time until it was undetected after 45 min in the rat kidney homogenate. The concentrations of the primary degradants exendin-4(15–39) and exendin-4(16–39) increased over the first 10 min, were relatively constant over the next 10 min, and then decreased until they were undetectable after 45 min. Similar concentrations of exendin-4(15–39) and exendin-4(16–39) were registered in the presence of this biological matrix. The rat kidney homogenate digested exendin-4 and its putative degradants to approximately 100% completion. The metabolic degradation of exendin-4 was lower in the rat kidney homogenate than in the rat liver homogenate. The highest concentration of the metabolite exendin-4(12–39) was found 50 min after incubation in the rat liver, and very low levels of exendin-4 and the exendin-4(12–39) fragment were detected in measurements taken up to 360 min after incubation.

## Discussion

Modifications of exendin-4 that prolong its biological half-life (e.g., stabilization towards exo- and endopeptidases) are necessary to improve its therapeutic utility. Because the pharmacological activity of exendin-4 is localized to tissues outside the central nervous system, study of the metabolism of exendin-4 in peripheral tissues, which include its primary target sites and major organs of elimination, is very important. A previous study [29] confirmed that the kidney is the major route of exendin-4 elimination and showed that the liver also plays as a major target organ for exendin-4 to exertits plasma loweringeffect in diabetic state rat. Similarly, our preliminary *in vitro* studies performed with rat/human plasma and heat-inactivated tissue homogenate control matrixes show that little degradation occurs in the circulation, and most peptide clearance occurs in target/distribution organs such as the kidney and liver. Therefore, in this study, we investigated the degradation of exendin-4 in homogenates from the rat liver and kidney. To identify the influence of these organs in terms of the extent of enzymatic degradation and the metabolic pattern of exendin-4, homogenates of the liver or kidney can be used for enzymatic metabolism studies [30, 31]. Tissue homogenates are a reliable biological system that can be used to investigate peptide metabolism in multiple organs and/or across species [32]. In tissue homogenates, the peptide substrate can be exposed to a mixture of enzymes from different metabolic compartments at concentrations higher than those found in subcellular fractions isolated from organs. No study has characterized the degradation of exendin-4 in homogenates of its known target organs (liver and kidney) [22]. The results of our study provide important information by describing the degradation pattern of exendin-4 *in vivo*.

In addition, to identify the specific peptidase involved in the metabolism/degradation of exendin-4, the peptide was further incubated in rat tissue homogenates in the presence of various general and specific protease inhibitors. Marked inhibition of exendin-4 degradation in the kidney was observed in the presence of 1,10-phenanthroline and DL-thiorphan, and PPACK, amastatin, PMSF, and bestatin showed inhibitory effects, which suggests that metalloproteases, endopeptidase 24–11, amino proteases, serine proteases, and DPP-IV are the predominant metabolizers of exendin-4. However, in the rat liver, PPACK and DL-thiorphan, which are inhibitors of DPP-IV and endopeptidase 24–11, respectively, did not significantly reduce exendin-4 metabolism, which implied that liver DPP-IV and endopeptidase 24–11 are not responsible for the cleavage of exendin-4, or alternatively suggested that liver DPP-IV and endopeptidase 24–11 in rats are not PPACK- and DL-thiorphan-sensitive. Thus, the primary metabolizers of exendin-4 in the rat liver homogenate include metalloproteases, amino proteases, serine proteases, and trypsin. Of all tested protease inhibitors, the metallopeptidase inhibitors most effectively increased the metabolic stability of exendin-4 in the tissue homogenates, implying that metallopeptidases are the primary degradative enzymes for exendin-4 in the kidney and liver. Recent advances in biological MS over the past decade now permit sensitive detection, accurate mass determination, and structural analysis of a wide range of biomolecules [33,34]. In this study, we used a combination of MALDI-TOF MS and LC-ESI-MS/MS to identify the fragments of the peptide exendin-4 that resulted from enzymatic degradation of exendin-4 in rat tissue homogenates. The products of metabolic cleavage of exendin-4 identified in both biological matrixes using both techniques were similar. However, some metabolic products found with MALDI-TOF MS were not detected with ESI-MS/MS in the same sample. These discrepancies can be explained by differences in the sample processing protocol used for each method. For MALDI-TOF MS analysis, exendin-4 native incubation solution was analyzed directly without further processing. The resulting spectra contained signals of the [M+1]^+^ ion of each peptide present in the incubation solution, and this direct analysis may have resulted in higher metabolite concentrations in comparison with those obtained after further processing of the incubation solutions before ESI-MS/MS analysis, e.g., precipitation and subsequent freeze drying. However, despite our use of the automated date-dependent acquisition (DDA) mode during HPLC introduction of peptide mixtures to the tandem mass spectrometer, double- and triple-charged peptides and low concentration levels of some degradation products made it difficult, and in some cases impossible, to positively determine the amino acid sequence using MS/MS technology. Because of these low levels, it is likely that additional degradation products were formed below the limits of detection. Although the time of analysis using MALDI-MS was reduced to only a few minutes, the accuracy of data generated from MALDI was lower than that obtained from ESI-MS/MS, particularly at lower masses. On the other hand, ESI-MS/MS, which has been successfully applied in proteolytic mapping/sequence analysis of peptides, provides selectivity and structural information [34,35] to validate the MALDI spectra. Thus, in the present study, a comprehensive application of MALDI-TOF and ESI-MS/MS enabled identification of peptide fragments that may arise from exendin-4 degradation in rat tissue.

**Fig 9:**
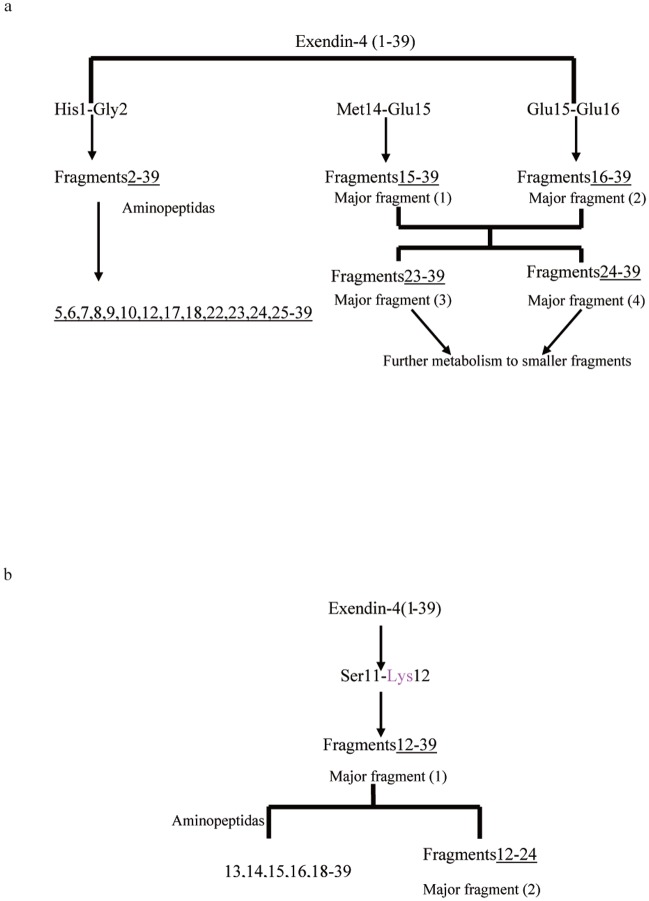
Putative degradation pathway for exendin-4 in rat (a) kidney and (b) liver homogenates.

As concluded from the analysis of MALDI-TOF and ESI-MS/MS data, the schemes for the exendin-4 degradation mechanism in the both rat tissue homogenates are shown in Fig. 9. In the kidney, the peptide is first cleaved by an endopeptidase, C-terminal to the Met and Glu-residues. The two major degradation products, exendin-4(15–39) and exendin-4 (16–39), are broken down further by exopeptidases. Inthe liver, the degradation pattern of exendin-4 was different with pronounced cleavage at positions (11–12). In addition, some minor differences in the secondary cleavage pattern were detected. Putative primary degradants and secondary degradants were further metabolized over time. The number of cleavage sites identified in the kidneywas higher than that identified in the liver. This may partly result from the differences in protease distribution among the tissues tested. It remains to be determined whether exendin-4(15–39), exendin-4(16–39), andexendin-4(12–39) fragments can be identified in vivo. By site-specific modification of peptides with long-chained polyethylene glycol (PEGylation) to protect cleavage sites, we designed and synthesized mono-PEGylated analogues of exendin-4, and found that mono-PEGylated exendin-4, with PEG linked near to the site of cleavage, showed a significantly longer half-life, a much lower average clearance rate, and substantial improvement in plasma exposure, in comparison with the native peptide *in vivo*, andalso exhibited stronger GLP-1 receptor agonist activity *in vitro*.

In this study, we used a combination of MALDI-TOF MS and LC-ESI-MS/MS to characterize the degradation of exendin-4 in rat kidney and liver homogenates *in vitro*. We found that the degradation of exendin-4 in the kidney and liver homogenates followed different patterns, and the primary cleavage sites of exendin-4 degradation in the rat kidney homogenate were located after AA-14, and-15, whereas those in the rat liver homogenate were located after AA-11. It is hoped that extending our knowledge of the degradative pathways of exendin-4 will lead to the development of peptide analogues with improved therapeutic potential to treat diabetes mellitus.

## Supporting Information

S1 Fig Matrix-assisted laser desorption/ionization time-of-flight mass spectrometry (MALDI-TOF MS) spectrum of degradation products from exendin-4 incubated in the rat kidney homogenate for 90 min. (TIF)
