# Correction: *In Vitro* Metabolic Stability of Exendin-4: Pharmacokinetics and Identification of Cleavage Products

**DOI:** 10.1371/journal.pone.0125296

**Published:** 2015-04-16

**Authors:** The PLOS ONE Staff

The information in the Funding section is incorrect. The correct Funding statement should read:

This work was supported by a grant from the National Natural Science Foundation of China (No. 81001469) and Chinese National Science & Technology Major Special Project on Major New Drug Innovation (2012ZX09301003-001-007). The funders had no role in study design, data collection and analysis, decision to publish, or preparation of the manuscript.
