# Supplementary figures and images for: In Vitro Metabolic Stability of Exendin-4: Pharmacokinetics and Identification of Cleavage Products
Source: PLoS One. 2015 Feb 27;10(2):e0116805. doi: 10.1371/journal.pone.0116805 (PMC4344207; doi:10.1371/journal.pone.0116805)

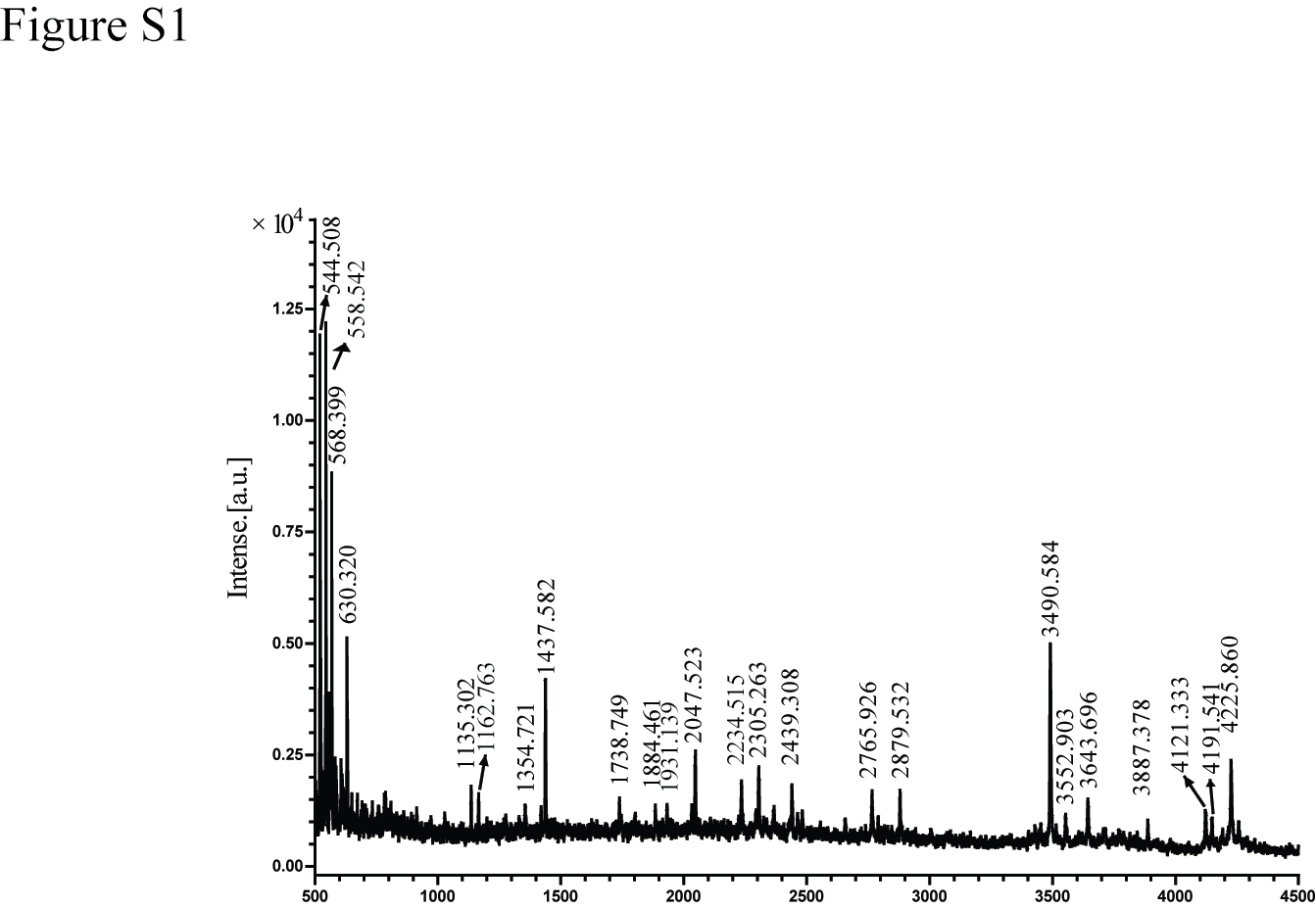

Supplement: S1 Fig — (TIF) [file pone.0116805.s001.tif]
